# Hispidulin Attenuates Cardiac Hypertrophy by Improving Mitochondrial Dysfunction

**DOI:** 10.3389/fcvm.2020.582890

**Published:** 2020-11-26

**Authors:** Yan Wang, Zengshuo Xie, Nan Jiang, Zexuan Wu, Ruicong Xue, Bin Dong, Wendong Fan, Gang Dai, Chen Chen, Jiayong Li, Hao Chen, Zi Ye, Rong Fang, Manting Choy, Jingjing Zhao, Yugang Dong, Chen Liu

**Affiliations:** ^1^Department of Cardiology, The First Affiliated Hospital of Sun Yat-sen University, Guangzhou, China; ^2^Department of Cardiology, The Second People's Hospital of Guangdong Province, Guangzhou, China; ^3^NHC Key Laboratory of Assisted Circulation (Sun Yat-sen University), Guangzhou, China; ^4^National-Guangdong Joint Engineering Laboratory for Diagnosis and Treatment of Vascular Diseases, Guangzhou, China; ^5^Department of Anesthesiology, The First Affiliated Hospital of Sun Yat-sen University, Guangzhou, China; ^6^Faculty of Medicine, St Vincent Clinical School, University of New South Wales, Sydney, NSW, Australia

**Keywords:** cardiac hypertrophy, hispidulin, mitochondrial dysfunction, heart failure, oxidative stress

## Abstract

Cardiac hypertrophy is a pathophysiological response to harmful stimuli. The continued presence of cardiac hypertrophy will ultimately develop into heart failure. The mitochondrion is the primary organelle of energy production, and its dysfunction plays a crucial role in the progressive development of heart failure from cardiac hypertrophy. Hispidulin, a natural flavonoid, has been substantiated to improve energy metabolism and inhibit oxidative stress. However, how hispidulin regulates cardiac hypertrophy and its underlying mechanism remains unknown. We found that hispidulin significantly inhibited pressure overload-induced cardiac hypertrophy and improved cardiac function *in vivo* and blocked phenylephrine (PE)-induced cardiomyocyte hypertrophy *in vitro*. We further proved that hispidulin remarkably improved mitochondrial function, manifested by increased electron transport chain (ETC) subunits expression, elevated ATP production, increased oxygen consumption rates (OCR), normalized mitochondrial morphology, and reduced oxidative stress. Furthermore, we discovered that Sirt1, a well-recognized regulator of mitochondrial function, might be a target of hispidulin, as evidenced by its upregulation after hispidulin treatment. Cotreatment with EX527 (a Sirt1-specific inhibitor) and hispidulin nearly completely abolished the antihypertrophic and protective effects of hispidulin on mitochondrial function, providing further evidence that Sirt1 could be the pivotal downstream effector of hispidulin in regulating cardiac hypertrophy.

## Introduction

Cardiac hypertrophy is a pathophysiological response to a series of predisposing conditions, including hypertension, coronary heart disease, valvular heart disease, and cardiomyopathy (1). Although cardiac hypertrophy is the compensatory response for preserving cardiac function initially, long-standing cardiac hypertrophy will progressively develop into heart failure (2, 3). Characterized by enlarged cell size and enhanced protein synthesis, cardiac hypertrophy is commonly accompanied by severe oxidative damage, which is associated with energy depletion (4). With a minute-to-minute high energy requirement, cardiac contraction and relaxation require sufficient energy supply to fulfill the workload demand (5). The mitochondrion is the primary organelle for energy production, mitochondrial dysfunction contributes to the development of heart failure (6). In hypertrophied hearts, the activity of mitochondrial oxidative phosphorylation complexes and ATP synthase are reduced, which leads to a decline in ATP production (7). In addition to abnormal mitochondrial morphology, decreased individual mitochondrial area and suppressed mitochondrial dynamics were observed (8). Meanwhile, impaired mitochondrial function results in the accumulation of reactive oxygen species (ROS) leakage from impaired electron transport chain (ETC). The aforementioned abnormalities could eventually lead to reduced energy production and enhanced oxidative stress of cardiomyocytes (9). Thus, maintaining the function of mitochondria is fundamental for protecting the heart from cardiac hypertrophy.

Hispidulin (4′,5,7-trihydroxy-6-methoxyflavone) is a natural flavonoid extracted from *Saussurea involucrata* (10). It has been demonstrated that hispidulin has anti-oxidant and metabolism modulation capacities. Hispidulin improves lipid metabolism by directly binding to peroxisome proliferator-activated receptor α (PPARα), a ligand-activated transcription factor involved in fatty acid metabolism, and activating PPARα *in vitro* (10). Hispidulin can activate PPARγ through the activation of adenosine 5′-monophosphate (AMP)-activated protein kinase (AMPK) and extracellular regulated protein kinases (ERK) signaling pathways, thereby suppressing the oncogenesis and metastasis of hepatocellular carcinoma both *in vitro* and *in vivo* (11). In addition, hispidulin has been proven with antioxidative properties predominantly by activating nuclear factor erythroid 2-related factor 2 (Nrf2) (12). Furthermore, hispidulin reverses cell apoptosis and mitochondrial dysfunction by activating the AMPK/Glycogen synthase kinase-3 (GSK3β) signaling pathway (13). Nevertheless, the effect of hispidulin on cardiac hypertrophy has not yet been investigated. Since energy insufficiency and oxidative damage are principal mechanisms of cardiac hypertrophy, we hypothesized that hispidulin might play a protective role against cardiac hypertrophy. The aim of our present study was to examine the effects of hispidulin on cardiac hypertrophy and explore the underlying mechanisms both *in vivo* and *in vitro*.

## Materials and Methods

### Reagent

Phenylephrine (PE) was purchased from Tokyo Chemical Industry (P0398). Hispidulin (SML0582), Dimethyl sulfoxide (DMSO) (D2650), and EX-527 (E7034) were obtained from Sigma-Aldrich (St. Louis, USA). With the purpose of detecting specific proteins by Western blotting, the following primary antibodies, obtained from Cell Signaling Technology (Danvers, MA, USA), were applied: anti-AMPK (Thr172) (#2531), antiphosphorylated AMPK (#2532), anti-Sirt1(D1D7) (#5490) antibody, anti-phosphorylated p38 (Thr180/Tyr182) (#4511), anti-p38 (#8690), anti-phosphorylated Akt (Ser473) (#4060), anti-Akt (#9272), anti-phosphorylated JNK1/2 (Thr183/Tyr185) (#4668), anti-JNK1/2 (#9252), anti-Tom20 (D8T4N) (#42406), anti-OPA1 (D6U6N) (#80471), anti-Mitofusin2 (D1E9) (#11925), and anti-GAPDH (#2118) antibodies. Anti-PGC-1α antibody (66369-lg) and secondary antibodies, including goat anti-rat IgG (H+L), horseradish peroxidase (HRP) conjugate (SA00001-15), goat anti-mouse IgG (H+L), and HRP conjugate (SA00001-1), were obtained from Protein-tech Group (Wuhan, China). The anti-vinculin antibody was purchased from Sigma-Aldrich (V9264).

### Animals

All animal experiments were conducted with compliance of the Guide for the Care and Use of Laboratory Animals published by the US National Institutes of Health and the Animal Care and Use Committees of the Sun Yat-sen University. Mice were raised at the Laboratory Animal Center of Sun Yat-sen University. Male C57BL/6J mice weighing 24-26 g (8-10 week old) were anesthetized through intraperitoneal injection of sodium pentobarbital (75 mg/kg) and endotracheally intubated. Mice then underwent descending aortic banding (AB) or sham surgery. The surgical procedure is briefly described as follows. After left thoracotomy under anesthesia, the descending aorta was separated. A 26-gauge needle was tied by a 7-0 silk suture ligature around the descending aorta to induce pressure overload in the AB group, and the aforementioned procedure was conducted without AB in the sham group (14). All the animals were randomly assigned to four groups: DMSO+sham, hispidulin+sham, DMSO+AB, hispidulin+AB, hispidulin (20 mg/kg/day, i.p., once daily) or equally volume of DMSO were injected in the mice intraperitoneally from 1 day before surgery to 1 week or 4 weeks post-surgery. The mice subsequently underwent echocardiography and were sacrificed 1 week or 4 weeks after surgery.

### Echocardiography

Four weeks after AB or sham surgery, the C57BL/6J mice underwent transthoracic echocardiographic examination using a commercially available echocardiographic system (VisualSonics Vevo 2100 with a 30-MHz transducer) after being sedated with inhalant anesthetics (1.5-2% isoflurane mixed with 100% O_2_) and placed in the supine position. The echocardiographic imaged were obtained by an experienced technologist who were blinded to the study design.

### RNA-Seq Analysis

RNA samples were collected from mice 4 weeks after AB or sham surgery. RNA isolation, library construction, and sequencing were performed on a BGISEQ-500 (Beijing Genomic Institution, www.genomics.org.cn, BGI). For the gene expression analysis, the fold changes were also estimated according to the fragments per kilobase of exon per million fragments mapped (FPKM) in each sample. The significance of different gene expression was defined by the following filter criteria: false discovery rate (FDR) ≤ 0.001 and log2-ratio ≥1. All original sequence datasets will be submitted to the GEO repository and GEO accession number is GSE154798.

### Transmission Electron Microscopy

Briefly, after euthanasia, the mouse heart was collected and fixed by immersing in 4% paraformaldehyde with 2.5% glutaraldehyde in 0.1 M sodium cacodylate. After fixation, tissues were cut from the heart, followed by sectioning into 70-nm slices using an ultramicrotome and stained with lead citrate. A Tecnai G2 spirit Twin transmission electron microscope (USA FEI) at 80 kV and 5,800× -62,500× direct magnification was used for ultrastructural detection of the mouse heart sections. Individual mitochondrial area and aspect ratios were measured with ImageJ software (National Institutes of Health, Maryland, USA).

### Adult Mouse Cardiomyocyte Culture

Adult mouse ventricular cardiomyocytes were cultured by using 8-week-old C57BL/6J mice. The heart were excised rapidly and fixed to Langendroff perfusion system, followed by perfusing through aorta with perfusion buffer (NaCl 113 mmol/l, KCl 4.7 mmol/l, KH2PO4 0.6 mmol/l, MgSO4 1.2 mmol/l, HEPES 10 mmol/l, NaHCO3 12mmol/l, KHCO3 10 mmol/l, Taurine 30 mmol/l, BDM 10 mmol/l, Glucose 5.5 mmol/l) for 5 min and with collagenase buffer (30 mg Type II collagenase diluted with 30 ml perfusion buffer) for 20 min. After that, hearts were transferred to the 100-mm culture dishes which were filled with collagenase buffer. After removing blood vessels, connective tissue and atria, the rest heart tissue were chopped completely and dipped gently. Then, all suspensions and tissues were transferred to stop buffer and mixed thoroughly, followed by filtration with 100 μm cell strainer, centrifuge at 300 rpm for 5 min and re-suspension. The aforementioned procedures were, repeated for three times. After that, cardiomyocytes were plated in 100 mm culture dishes with culture media and preplated for 1 h to remove fibroblasts. Then the cell suspension was collected and plated in six-well plates with culture media. After incubated for 24 h, mouse adult cardiomyocytes were treated with hispidulin for 24 h.

### Primary Cardiomyocyte Culture

Neonatal rat cardiomyocytes were prepared as described previously (15). Briefly, neonatal rat ventricular cardiomyocytes (NRVMs) were cultured by using 1-2-day-old Sprague Dawley rats. The left ventricles were cut into 2 mm^3^ pieces and digested with 0.125% trypsin (20885552, Gibco) at 37°C for 5 min. After discarded the supernatant, the remaining tissue was digested with 0.05% type I collagenase (17018029, Gibco) at 37°C for 2.5 h and neutralized with an equal volume of DMEM containing 10% Newborn Calf Serum (NBS). The cell suspensions were collected and the rest of the tissue was redigested in 0.125% trypsin at 37°C for another 5 min. The suspension was collected. All suspensions were collected and centrifuged thrice at 510 rpm for 8 min. The suspensions were separated and dipped in the D-Hanks solution. After this step was repeated twice, the isolated cells were resuspended in DMEM containing 10% NBS and preplated for 1 h to remove fibroblasts. The cardiomyocytes were then plated in six-well plates at a density of 0.8 × 10^6^ cells/well with DMEM/F12 containing 10% NBS and 0.1 mM 5-bromodeoxyuridine (BrdU). Forty-eight hours after seeding, the cardiomyocytes were starved with a serum-free medium for 24 h. Then, the cardiomyocytes were treated with hispidulin for 2 h before PE treatment and exposed to 50 μM PE for 24 h to induce hypertrophy.

### RNA Isolation and Quantitative Real-Time PCR (qPCR)

Total RNA was extracted from cardiomyocytes and heart tissues with TRIzol (T9424, Sigma-Aldrich), and single-strand cDNA was prepared by using a RevertAid First Strand cDNA Synthesis Kit (K1622, Thermo Fisher) following the manufacturer's instructions. RNA levels were detected by real-time PCR using a LightCycler480 thermal cycler (Roche, Germany). PCR amplification was performed as follows: 95°C for 5 min, 39 cycles of 95°C for 10 s, 60°C for 20 s, and 72°C for 20 s. The primers used are summarized in Table 1.

**Table 1:** Sequences of the primers.

**Target**	**Forward (5′-3′ orientation)**	**Reverse (5′-3′ orientation)**
ANP (rat)	TGAGCCGAGACAGCAAACATC	AGGCCAGGAAGAGGAAGAAGC
BNP (rat)	CAGCTCTCAAAGGACCAAGG	TAAAACAACCTCAGCCCGTC
GAPDH (rat)	ACAGCAA CAGGGTGGTGGAC	TTTGAGGGTGCAGCGAACTT
SOD1 (rat)	AGGGCGTCATTCACTTCGAG	CCTCTCTTCATCCGCTGGAC
CATA (rat)	GCCAATTTCGACAGGGAACG	CGCTCATGGATAACGGTGGA
MnSOD (rat)	ACCGAGGAGAAGTACCACGA	TGGGTTCTCCACCACCCTTA
Prx-3 (rat)	CCTGGATCAACACGCCAAGA	GCAATGCCAGCACTTTCCAA
Mitofusin 1 (rat)	CAAAGAAGGCCATCACTGCG	TCCGATCAAGTTCCGGGTTC
Mitofusin 2 (rat)	TCAGTAGCCAATCTGGACCTG	AAGGGACCTTCACAGGTTGG
OPA1 (rat)	GCCCTTCCCAGTTCAGAAGA	GGTGTACCCGCAGTGAAGAA
Drp1 (rat)	TGGAAAGAGCTCAGTGCTGG	CAACTCCATTTTCTTCTCCTGTTGT
Fis1 (rat)	ACCCAAGCGTGCTTTCTGTA	TCATCCCTTACCACGCAACC
Mff (rat)	GCCCAGGGGATAAAGGTGAC	AGTGGGAGAAGGAAATGCCG
ANP (mouse)	ACCTGCACCACCTGGAGG	CCTTGGCTGTTATCTTCGGTACCG
BNP (mouse)	GCTGTAACGCACTGAAGTTGT	ATCACTTCAAAGGTGGTCCCAG
GAPDH (mouse)	GTTGTCTCCTGCGACTTCAAC	GCTGTAGCCGTATTCATTGTCA
SOD1 (mouse)	TTGGCCTGTGGAGTGATTGG	CAGTTTAATGGTTTGAGGGTAGCA
CATA (mouse)	ATGGAAGGACCGTGTTTGGTT	CGCTGGCGCTTTTATTGTTACT
MnSOD (mouse)	GAGAGCAGCGGTCGTGTAAA	AGCCTCGTGGTACTTCTCCT
Prx-3 (mouse)	TCGGTATCTCCGCCTATCGT	GAGGACCAGAGCAACCTTCC
Atp2a2 (mouse)	GGAACCTTTGCCGCTCATTT	AGGGAGCAGGAAGATTTGGTG
Atp5b (mouse)	AGCTCTGACTGGTTTGACCG	GCCCAATAAGGCAGACACCT
Atp5j1 (mouse)	CTCCTGTTTGGCTTCTGTCTCA	GCCATCGCAAGGCACTATGG
Atp5f1 (mouse)	TCCAGGGGTATTACAGGCAAC	CAGCCCAAGACGCACTTTTC
Atp5a (mouse)	GGCAACCACAAGGTCGATTC	CGGACGACTGGCACAAAATG
Atp5o (mouse)	TGTTCAGGTCTACGGCATCG	GGTGCGCTTGATGTAGGGAT
Usmg5 (mouse)	CGTCGCTAGGTTTGTTGAAGG	GCCATCACTTTCTGCACCAG
Ndufa1 (mouse)	GAGAGGTAAAGCCGGGTCAC	GACCAAGCACACCCCCATAA
Ndufa4 (mouse)	AAACATGCTCCGCCAGATCC	GTGCCAAGCGCATCACATAC
Ndufa3 (mouse)	TCAACAAGGCCACACCCTAC	ACAGGTTCTTCAGCCAGTCC
Ndufa5 (mouse)	GACACTCCACACGAGAGGC	CGCCTTGACCATATCCAGCTT
Ndufs1 (mouse)	GGTTTTGAGGGGTCAGGGAG	CCAGTCAAGGGCAAGAGGAG
Uqcrb (mouse)	TCTCAGGTCAAAATGGCGGG	TCAGGTCCAGGGCTCTCTTA
Sucla2 (mouse)	GCCGCCCAGGATCAAAGGAT	CTGCCCTTTTCTCCAGTTTGC
Uqcrc2 (mouse)	GTCCTCCGACCCCATCTTG	TCATGGCTCTAGCAGTTGCC

### Western Blotting Analysis

Western blotting was performed as described in our previous study (14). Briefly, cardiomyocytes were harvested on ice with cold cell lysis buffer which contained 1 mM PMSF (#8553S, CST) and 1× phosphatase inhibitor (#5870, CST). The heart homogenates were obtained as follows: hearts were harvested and homogenized using lysis buffer in an ice-cold Dounce tissue grinder, and the tissue was subjected to ultrasound three times for dissociation. After centrifugation at 10,000 g for 10 min, the supernatant was collected for western blotting. Protein concentrations were measured using a Pierce™ BCA Protein Assay Kit (23250, Thermo Fisher). Equal amounts of protein (20 μg) for each sample were prepared and size-separated by SDS-PAGE, transferred to 0.45 μm PVDF membranes (IPaVH00010, Millipore) and blocked with 1× Tris buffered saline with Tween (TBST) containing 5% bovine serum albumin (BSA) (#9997, CST) for 1 h. After being incubated overnight at 4°C, primary antibodies were diluted as follows: anti-Sirt1, anti-phosphorylated JNK1/2, anti-JNK1/2, anti-phosphorylated p38, anti-p38, anti-phosphorylated Akt, anti-Akt, anti-PGC-1α, anti-AMPK, and anti-phosphorylated AMPK antibody were diluted as 1:1,000 using 5% 1 × TBST containing 5% BSA; anti-GAPDH and anti-Vinculin antibody were diluted as 1:10,000. Then the second antibody were subsequently incubated at room temperature for 1 h, and the signals were visualized with Pierce™ ECL Western Blotting Substrate (32106, Thermo Fisher). The signals were quantified using Quantity One software (Bio-Rad, CA, USA). GAPDH and vinculin were used as internal controls.

### Immunofluorescence Staining

Immunofluorescent staining was performed as previously described (14). Primary antibodies including rabbit polyclonal anti-troponin I, were diluted at 1:50, (sc-133117, Santa Cruz), and the immune complexes were detected with Cy3-conjugated secondary antibodies in a 1:100 dilution. The nuclei were stained with DAPI (40,60-diamidino-2-phenylindole, 0.5 mg/ml, Sigma-Aldrich, D9542). The images were captured at 400× with a LSM-780 confocal microscope (Zeiss, Oberkochen, Germany).

### Measurement of the Cell Surface Area

Twenty-four hours after PE treatment, the cardiomyocytes were washed three times with the D-Hanks buffer and then fixed with 4% paraformaldehyde for 30 min at room temperature. Cardiomyocyte surface area measurement was conducted using Image-Pro Plus software (Media Cybernetics, Crofton, MA, USA) as previously described (14).

### ATP Detection Assay

Twenty-four hours after PE treatment or 4 weeks after surgery, cardiomyocyte lysates or tissue homogenates were collected, respectively, being kept on ice throughout the experiment. ATP detection was conducted using a Molecular Probes' ATP Determination Kit (A22066, Thermo Fisher). The reaction was initiated by dispensing Luciferase reagent into all samples. The ATP concentration was measured by a bioluminescence assay (emission maximum ~560 nm at pH 7.8) and adjusted according to the protein concentrations of each sample. The results were presented as nmol/mg protein.

### Seahorse Mitochondrial Oxygen Consumption Rate (OCR) Assay

OCR detection of cardiomyocytes was conducted with the Seahorse XFe 24 analyzer (Agilent, Santa Clara, USA). Cardiomyocytes were seeded at a density of 1.5 × 10^4^/well in a special Seahorse 24-well plate (102342-100, Seahorse). After treating with PE for 24 h, the medium was replaced with XF medium containing 10 mM D-(+)-glucose (G7021, Sigma-Aldrich), and 1 mM sodium pyruvate (11360070, Thermo Fisher). The plates were placed in a CO_2_-free 37°C incubator for an hour before the measurement. A Seahorse XF Cell Mito Stress Test Kit (103015-100, Seahorse) was used for detection. The basal respiration of the NRVMs was detected, and ATP-linked OCR was analyzed after treatment with 1 μM oligomycin (ATP synthase inhibitor). Then, the uncoupling agent carbonyl cyanide 4-(trifluoromethoxy) phenylhydrazone (FCCP) 0.5 μM was injected to detect maximal respiration. Finally, rotenone and antimycin A (AR) were used to abolish mitochondrial respiration by inhibiting complexes I and III, respectively. Basal respiration was the average of the first three measurements. The mitochondrial-specific OCR (mito-OCR) was calculated by subtracting OCR values after the addition of AR from basal respiration. The ATP-linked OCR was measured by subtracting OCR values after oligomycin injection from basal OCR values. Maximal respiration was taken as the average of three measurements after FCCP injection. Reserve capacity was defined as the difference between maximal respiration and basal OCR.

### ROS Staining

ROS staining was performed as described in our previous study (16). DHE staining was conducted in frozen sections of the heart to detect ROS levels. Fresh hearts were harvested, embedded in optimum cutting temperature compound (OCT), and cut into 5 μm-thick frozen sections. The sections were stained with DHE (1 μm) for 20 min at 37°C. Fluorescence images were taken by a fluorescence microscope (DMi8, LEICA, German). The entire process was protected from light. Fluorescence intensity was measured using ImageJ software. DCF-DA staining was conducted in cardiomyocytes following the manufacturer's instructions. After treated with PE, cardiomyocytes were washed thrice with the D-Hanks buffer and incubated with 5 μM DCF-DA for 20 min at 37°C. Fluorescence images were taken using a fluorescence microscope (DMi8, LEICA, German). Fluorescence intensity was detected by flow cytometry (Gallios, Beckman Coulter, California, USA). Mitosox-Red staining was conducted in cardiomyocytes to detect mitochondrial ROS levels. After treating with PE for 24 h, cardiomyocytes were washed three times with the D-Hanks buffer and incubated with 5 μM Mitosox-Red for 20 min at 37°C. Fluorescence images were obtained using a fluorescence microscope (DMi8, LEICA, German). Fluorescence intensity was measured using ImageJ software.

### Statistical Analysis

All data are expressed as mean ± SE. The differences between means were evaluated using one-way or two-way ANOVA, followed by the least significant difference (LSD) posttest. Statistical significance was defined as a *p* < 0.05. All statistical analyses were performed using SPSS13.0 software (PASW, SPSS Inc., Chicago, IL, USA).

## Results

### Hispidulin Attenuated Pressure Overload-Induced Cardiac Hypertrophy in Mice

We evaluated the regulatory effect of hispidulin on pressure overload-induced cardiac hypertrophy in C57BL/6J mice. A hypertrophic model was conducted by aortic banding (AB) surgery, and sham surgery was used as a control. From 1 day before surgery to 4 weeks post-surgery, mice were treated with hispidulin (20 mg/kg/day, i.p., once daily), and DMSO was used as solvent control. Four weeks after surgery, the mice were sacrificed, and the cardiac hypertrophy phenotype was evaluated. Whether the hypertrophic models had been successfully established was confirmed by three criteria: (i) increased heart weight (HW/BW, HW/TL) and enlargement of the heart (left ventricular posterior wall thickness in diastole (LVPWD), left ventricular end-diastolic dimension (LVEDD), and left ventricular end-systolic dimension (LVESD) on echocardiography); (ii) worsening cardiac function (ejection fraction (EF%) and fractional shortening (FS%) on echocardiography); (iii) increased hypertrophic biomarkers (atrial natriuretic peptide (ANP), B-type natriuretic peptide (BNP), and hypertrophic signaling pathways).

**Figure 1:**
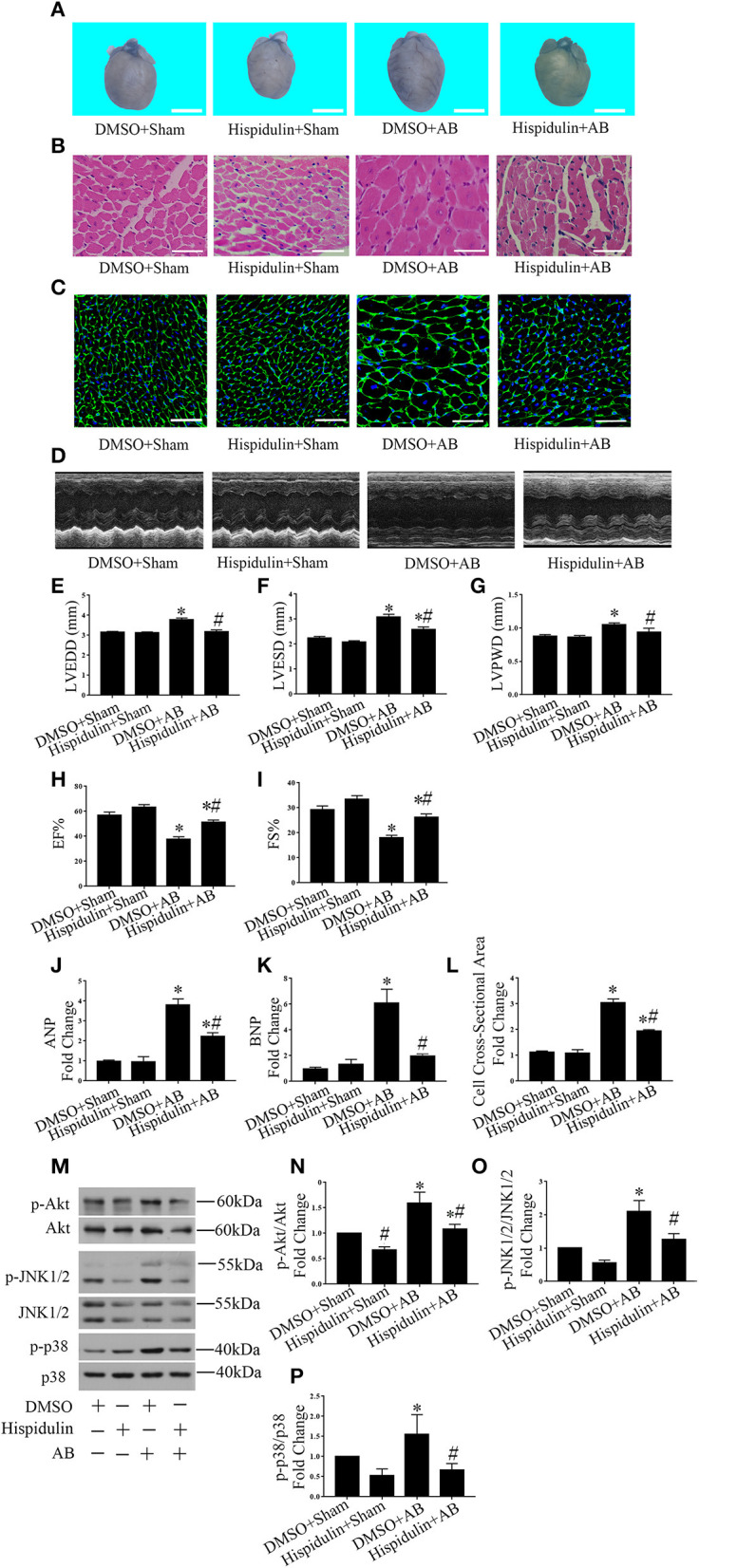
Hispidulin suppressed cardiac hypertrophy *in vivo*. Hispidulin was intraperitoneally injected into mice 1 day before surgery through 4 weeks after surgery. **(A)** Images of hearts harvested 4 weeks after surgery. The scale bar reflects 2 mm. **(B)** H&E staining of heart paraffin sections 4 weeks after surgery. Scale bar reflects 50 μm. **(C)** WGA staining of mouse heart sections 4 weeks after surgery. Scale bar reflects 50 μm. **(D)** Echocardiographic images of the hearts in each group. **(E–H)** Histograms of LVEDD **(E)**, LEVSD **(F)**, LVPWD **(G)**, EF% **(H)**, and FS% **(I)** (*n* = 8–12). **(J,K)** The mRNA levels of ANP and BNP in each group. GAPDH served as an internal control (*n* = 4–6). **(L)** Quantification of the heart cross-sectional area of each group. At least 50 cells were counted in each heart (*n* = 3–5). **(M)** Representative immunoblots of hypertrophic pathways, including Akt, JNK1/2, and p38. **(N–P)** Quantitative analysis of phosphorylated and total Akt **(N)**, JNK1/2 **(O)**, p38 **(P)** expression *in vivo* (*n* = 3–5). Data are expressed as the mean ± SE. Significance of the difference between DMSO+Sham and DMSO+AB or between Hispidulin+Sham and Hispidulin+AB: ^*^p < 0.05; significance of the difference between DMSO+Sham and Hispidulin+Sham or between DMSO+AB and Hispidulin+AB: ^#^*p* < 0.05 (n represents the number of independent experiments).

Importantly, we discovered that hispidulin significantly attenuated AB-induced cardiac hypertrophy. As shown in Figure 1A and Table 2, compared with the DMSO control, hispidulin reduced heart size, decreased the AB-induced increase in heart weight/body weight ratio (HW/BW), heart weight/tibia length ratio (HW/TL) by 17.49 and 21.71%, respectively. Moreover, hispidulin ameliorated AB-induced heart enlargement, manifested by decreased LVPWD, LVEDD, and LVESD (8.31, 14.50, and 15.10%, respectively) (Figures 1D–G). HE and WGA staining confirmed that hispidulin reduced the AB-induced increase in cross-sectional area (Figures 1B,C,L). We further confirmed this significant phenotype in the levels of hypertrophic biomarkers, manifested by decreased expression of ANP and BNP mRNA (37.50 and 66.67%, respectively) after hispidulin treatment in AB surgery (Figures 1J,K). Prohypertrophic pathways were detected to evaluate the effect of hispidulin on cardiac hypertrophy. As shown in Figures 1M–P, AB significantly activated protein kinase B (Akt), c-Jun N-terminal protein kinase1/2 (JNK1/2) and protein kinase 38 (p38) pathways, and hispidulin treatment suppressed the phosphorylation levels of Akt, JNK1/2 and p38 pathways. Furthermore, we elucidated that hispidulin not only attenuated cardiac hypertrophy and ventricular enlargement but also improved cardiac function. As shown in Figures 1H,I, the hispidulin+AB group exhibited elevated EF% and FS% of the heart compared with the AB group. Taken together, the data indicated that hispidulin attenuated pressure overload-induced cardiac hypertrophy and improved cardiac function.

**Table 2:** The effects of hispidulin on cardiac hypertrophy induced by AB surgery.

	**DMSO Sham**	**Hispidulin Sham**	**DMSO AB**	**Hispidulin AB**	**Hispidulin AB+EX527**
**Number**	***n* =11**	***n* = 11**	***n* = 17**	***n* = 16**	***n* = 5**
BW (g)	25.45 ± 1.70	25.65 ± 1.95	25.05 ± 1.95	24.51 ± 1.12^*^^#^	22.82 ± 1.06^*^^&^
HW/BW (mg/g)	5.14 ± 0.41	4.74 ± 0.23	7.72 ± 1.53^*^	6.37 ± 0.73^*^^#^	8.55 ± 0.91^*^^&^
LW/BW (mg/g)	6.01 ± 0.76	6.01 ± 0.54	7.81 ± 2.13^*^	6.52 ± 1.01^#^	8.20 ± 1.94^*^
HW/TL (mg/mm)	6.84 ± 0.91	6.17 ± 0.63	9.81 ± 1.60^*^	7.68 ± 0.81^*^^#^	10.01 ± 0.87^*^^&^
LW/TL (mg/mm)	7.99 ± 1.18	7.78 ± 0.66	9.96 ± 2.36^*^	7.93 ± 1.34^#^	9.50 ± 2.04

*AB, aortic banding; BW, body weight at 4 weeks after surgery; HW, heart weight; LW, lung weight; TL, tibia length*.

*Data was represented as the mean ± SD, n = 5–17. Significance of the difference between DMSO+Sham and DMSO+AB or between Hispidulin+Sham and Hispidulin+AB or between Hispidulin+Sham and Hispidulin+AB+EX527*:

* *p < 0.05; significance of the difference between DMSO+AB and Hispidulin+AB*:

# *p < 0.05; significance of the difference between Hispidulin+AB and Hispidulin+AB+EX527*:

& *p < 0.05 (n represents the number of independent experiments)*.

### Hispidulin Inhibited the Hypertrophic Response of Cardiomyocytes *in vitro*

**Figure 2:**
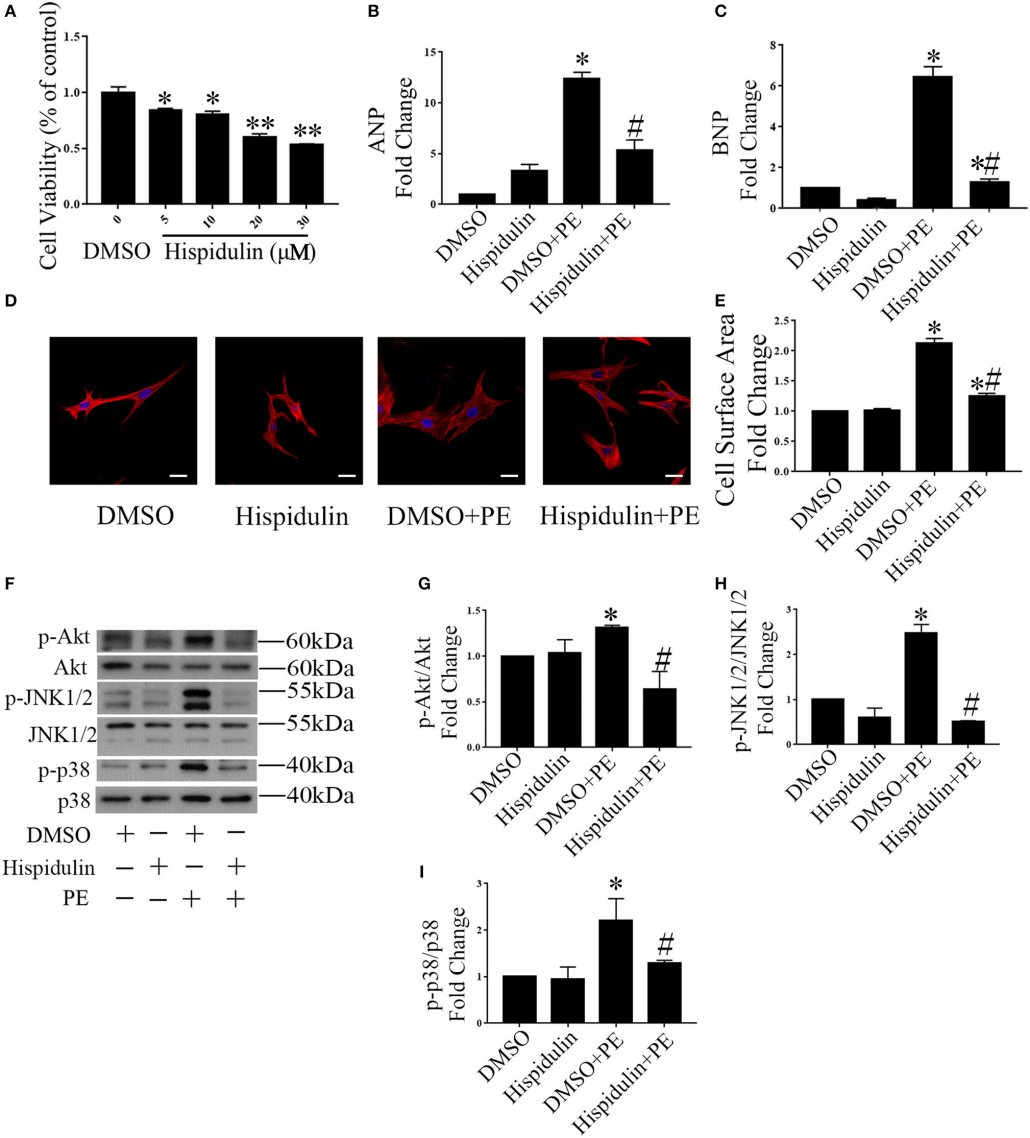
Hispidulin suppressed cardiac hypertrophy *in vitro*. **(A)** MTT assay was conducted to analyze cell viability after treatment with hispidulin (*n* = 3). Data are expressed as the mean ± SE. Significance of the difference between DMSO and Hispidulin: ^*^p < 0.05, ^**^p < 0.01. **(B,C)** After 2 h of treatment with hispidulin or DMSO, cardiomyocytes were treated with or without PE for 24 h. The mRNA expression of ANP **(B)** and BNP **(C)** was measured by qPCR. GAPDH was used as an internal control (*n* = 3). **(D)** Immunofluorescence staining was conducted using anti-troponin I antibody (1:50) and Cy3-conjugated secondary antibodies (1:100). The nuclei were stained with DAPI (0.5 mg/ml). Scale bar reflects 20 μm. **(E)** Cell surface area was measured 24 h after PE treatment (*n* = 3). **(F)** Representative immunoblots of hypertrophic pathways, including Akt, JNK1/2, and p38. **(G–I)** Quantitative analysis of phosphorylated and total Akt **(G)**, JNK1/2 **(H)**, and p38 **(I)** expression *in vitro* (*n* = 3). Data are expressed as the mean ± SE. Significance of the difference between DMSO and DMSO+PE or between Hispidulin and Hispidulin+PE: ^*^p < 0.05; significance of the difference between DMSO+PE and Hispidulin+PE: ^#^*p* < 0.05 (n represents the number of independent experiments).

To further determine whether hispidulin regulates cardiac hypertrophy, we tested the effect of hispidulin on cardiomyocyte hypertrophy *in vitro*. First, an MTT assay was conducted to analyze cell viability after treatment with hispidulin. As shown in Figure 2A, cardiomyocytes were treated with different concentrations of hispidulin ranging from 0 to 30 μM, and cell viability exceeded 80% when the concentration of hispidulin was not greater than 10 μM. Then, cardiomyocytes were treated with hypertrophic agonist phenylephrine (PE) in the presence or absence of hispidulin. After 24 h, the cells were harvested, and the hypertrophic phenotype was analyzed. As shown in Figures 2B,C, PE-treated cardiomyocytes showed increased ANP and BNP, whereas the hypertrophic biomarkers were markedly reduced in hispidulin-treated cardiomyocytes at a concentration of 10 μM. Consistently, hispidulin treatment significantly inhibited PE-induced cell size enlargement (Figures 2D,E). Hypertrophic stimuli are known to increase the phosphorylation of prohypertrophic signaling pathways, including Akt, JNK1/2, and p38. As shown in Figures 2F–I, hispidulin treatment significantly abolished PE-induced activation of Akt, JNK1/2, and p38. Therefore, these results further corroborated that hispidulin could protect against cardiac hypertrophy.

### Hispidulin Restored Mitochondrial Oxidative Phosphorylation in Mice

**Figure 3:**
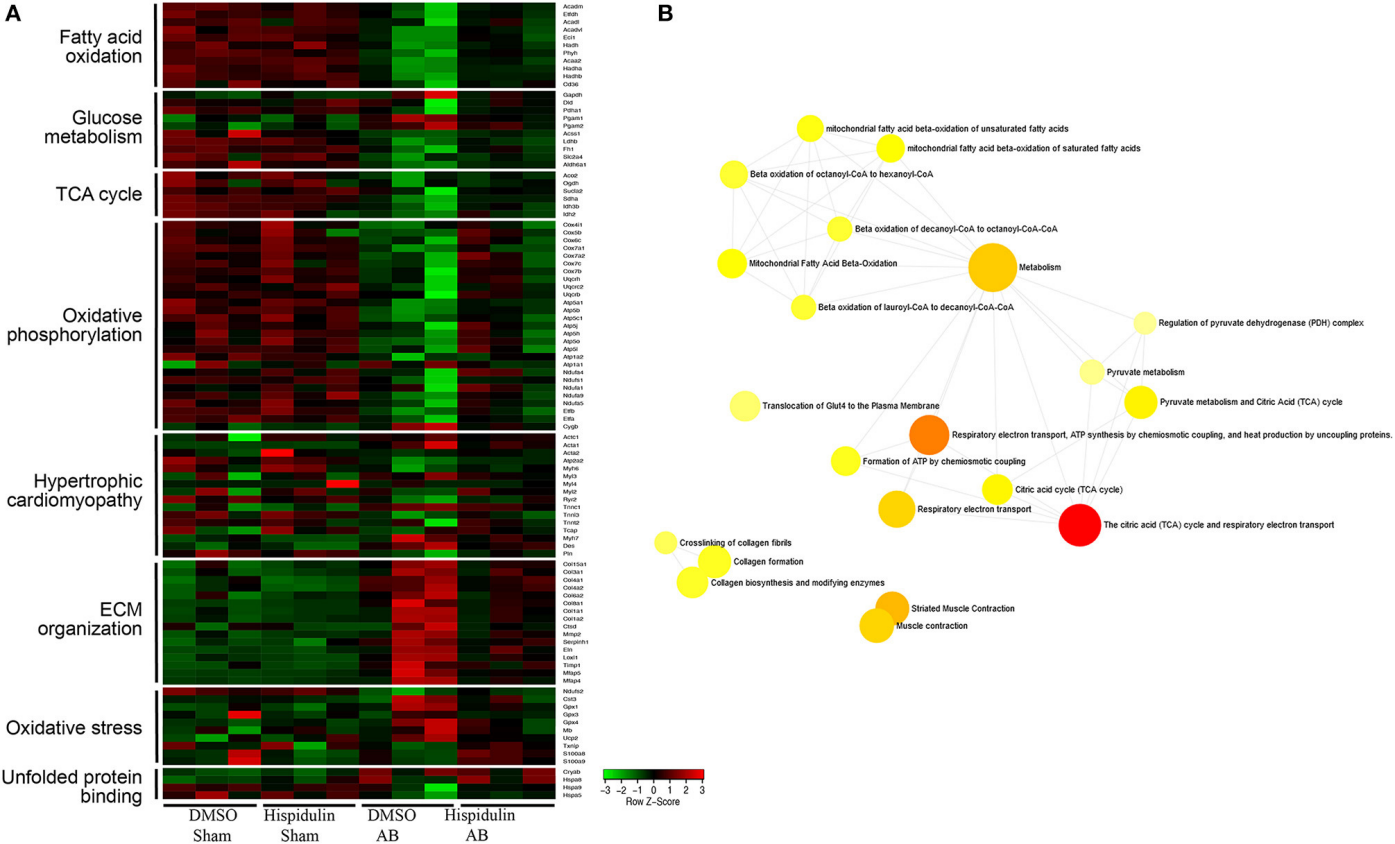
RNA-sequencing analysis was conducted on mouse hearts 4 weeks after surgery. **(A)** A heat map representing the expression of genes involved in fatty acid oxidation, glucose metabolism, TCA cycle, oxidative phosphorylation, hypertrophic cardiomyopathy, ECM organism, oxidative stress, and unfolded protein binding in each group (*n* = 3). Expression levels are showed as a color code ranging from green (low expression) to red (high expression). **(B)** Gene ontology analysis was performed of DMSO+AB and Hispidulin+AB (*n* = 3).

**Figure 4:**
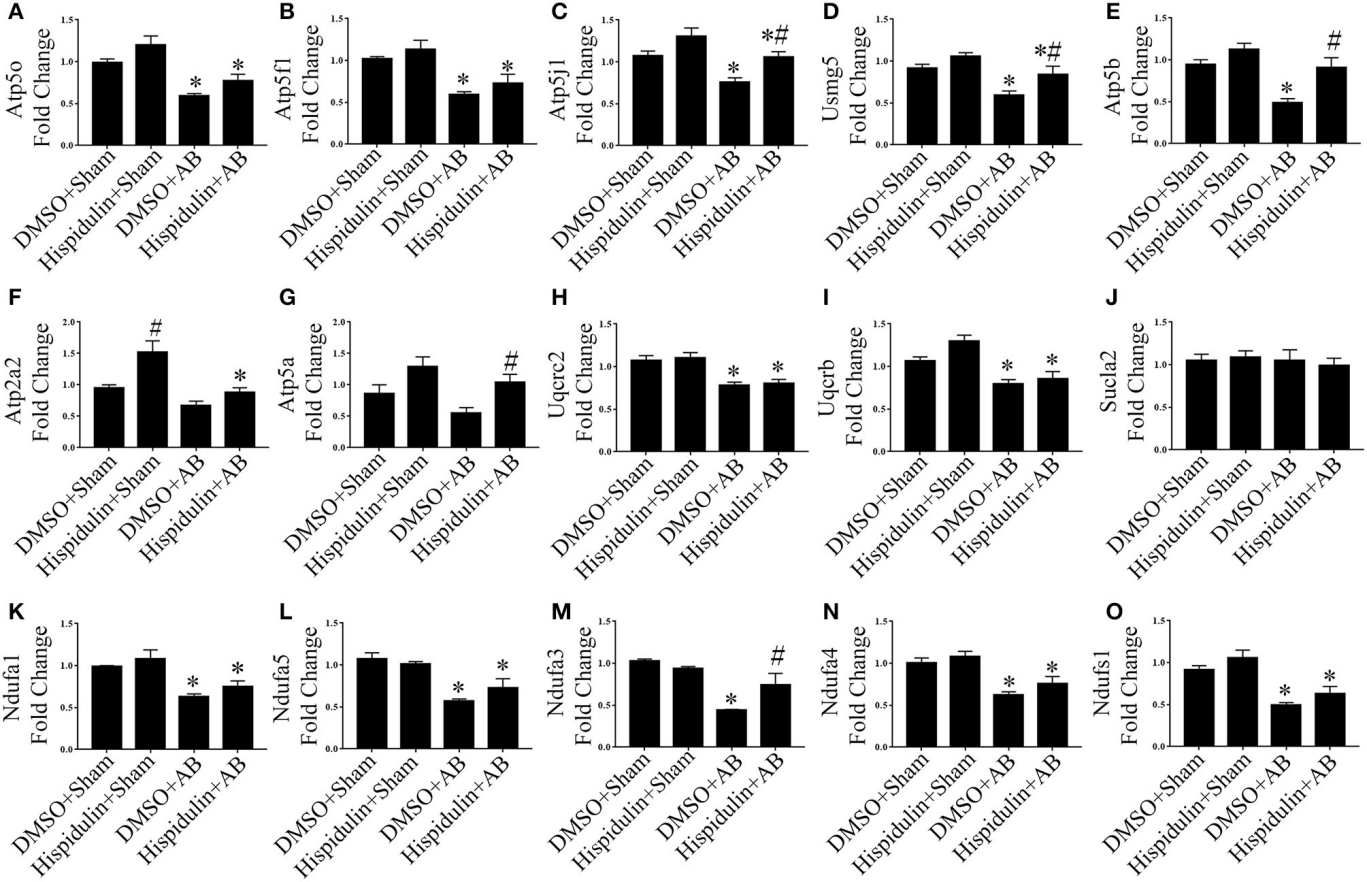
Hispidulin restored mitochondrial oxidative phosphorylation in mice. **(A–O)** Relative gene expression levels of oxidative phosphorylation complex subunits including Atp5o **(A)**, Atp5f1 **(B)**, Atp5j1 **(C)**, Usmg5 **(D)**, Atp5b **(E)**, Atp2a2 **(F)**, Atp5a **(G)**, Uqucrc2 **(H)**, Uqucrb **(I)**, Sucla2 **(J)**, Ndufa1 **(K)**, Ndufa5 **(L)**, Ndufa3 **(M)**, Ndufa4 **(N)**, and Ndufs1 **(O)** were determined by qPCR (*n* = 3–4). Data are expressed as the mean ± SE. Significance of the difference between DMSO+Sham and DMSO+AB or between Hispidulin+Sham and Hispidulin+AB: ^*^p < 0.05; significance of the difference between DMSO+Sham and Hispidulin+Sham or between DMSO+AB and Hispidulin+AB: ^#^*p* < 0.05 (n represents the number of independent experiments).

To further explore mRNA expression profiles in DMSO, AB, and hispidulin treatment, RNA-sequencing analysis was conducted in mice 4 weeks after surgery. As shown in Figure 3, after comparing of DMSO+AB group and hispidulin+AB group, we found that 113 genes were significantly differentially expressed. Gene ontology and cluster analysis indicated that alternated genes were enriched in fatty acid oxidation, glucose metabolism, tricarboxylic acid (TCA) cycle, oxidative phosphorylation, hypertrophic cardiomyopathy, ECM organism, oxidative stress and unfolded protein binding. Genes involved in hypertrophic cardiomyopathy such as Myh6 and Acta2, cardiac fibrosis such as Mmp2 and Col3a1, were all highly up-regulated by AB surgery, and hispidulin treatment significantly down-regulated these genes. Fatty acid oxidation (Acadm, Etfdh, Acadl, Acadvl, Eci1, Hadh, Phyh, Acaa2, Hadha, Hadhb, Cd36), glucose metabolism (Dld, Pdha1, Pgam1, Pgam2, Acss1, Ldhb, Fh1, Slc2a4, Aldh6a1), TCA cycle (Aco2, Dld, Fh1, Ogdh, Sucla2, Sdha, Idh3b, Idh2) were up-regulated by hispidulin in cardiac hypertrophy. Besides, pressure overload suppressed the expression of subunits of ATP synthase enzyme, complex I, and complex II, while hispidulin treatment significantly restored the mRNA expressions of these subunits, which indicated that hispidulin improved the capacity of mitochondrial oxidative phosphorylation. Corresponding quantitative PCR results were showed in Figures 4A–O. Hispidulin significantly up-regulated the expression of Atp5j1, Usmg5, Atp5b, Atp5a, Ndufa3 after AB surgery. Hispidulin also upregulated the expression of Atp5o, Atp5f1, Atp2a2, Uqcrb, Ndufa1, Ndufa5, Ndufa4, Ndufs1 after AB surgery slightly, but the alternation was insignificant.

### Hispidulin Maintained Mitochondrial Function Both *in vivo* and *in vitro*

**Figure 5:**
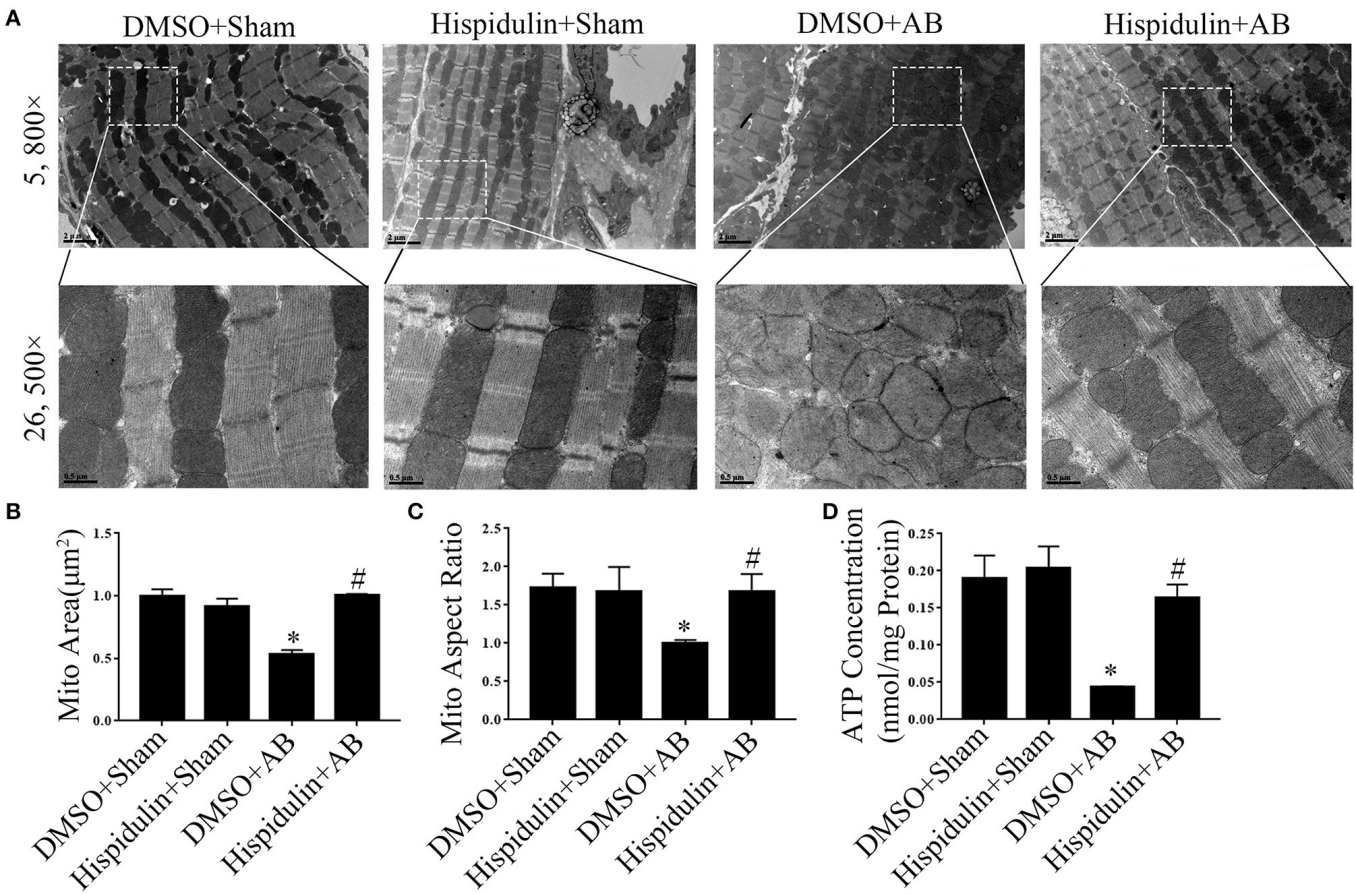
Hispidulin maintained mitochondrial ATP production and structural normality in mice. **(A)** Transmission electron microscopy was used for ultrastructural detection of mouse heart sections in each group. Scale bar reflects 0.5 μm (5800×) and 2 μm (26500×). **(B,C)** Quantification of individual mitochondrial areas and aspect ratios. Five images of each heart were evaluated (*n* = 3). **(D)** ATP concentrations in heart tissues were measured by a bioluminescence assay immediately after the hearts were harvested (*n* = 3). Data are expressed as the mean ± SE. Significance of the difference between DMSO+Sham and DMSO+AB: ^*^p < 0.05; significance of the difference between DMSO+AB and Hispidulin+AB: ^#^*p* < 0.05 (n represents the number of independent experiments).

**Figure 6:**
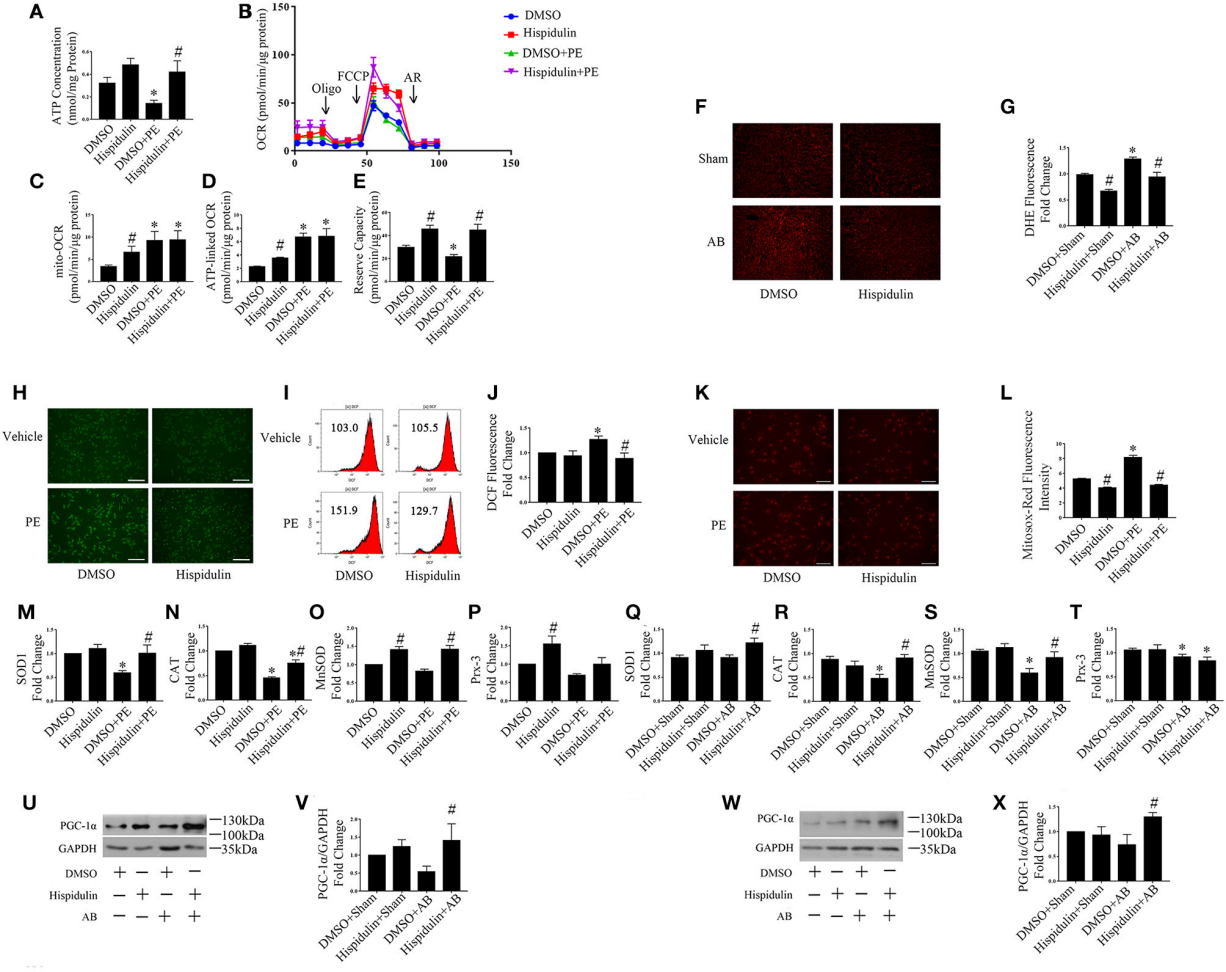
Hispidulin improved mitochondrial dysfunction. **(A)** ATP concentration in cardiomyocytes was measured by a bioluminescence assay (*n* = 3). **(B)** Mitochondrial respiration in cardiomyocytes was measured using a Seahorse XFe 24 analyzer (*n* = 3). Quantification analysis of mito-OCR **(C)**, ATP-linked OCR **(D)**, and reserve capacity **(E)** in each group (*n* = 3). Significance of the difference between DMSO and DMSO+PE or between Hispidulin and Hispidulin+PE: ^*^p < 0.05; significance of the difference between DMSO and Hispidulin or between DMSO+PE and Hispidulin+PE: ^#^*p* < 0.05. **(F)** Representative images of DHE-stained heart frozen sections. **(G)** Fluorescence intensity was measured by ImageJ software (*n* = 3). Significance of the difference between DMSO+Sham and DMSO+AB: ^*^p < 0.05; significance of the difference between DMSO+Sham and Hispidulin+Sham or between DMSO+AB and Hispidulin+AB: ^#^*p* < 0.05. **(H)** Representative images of DCF-DA-stained cardiomyocytes. Scale bar reflects 200 μm. **(I,J)** Fluorescence intensity was measured by flow cytometry (*n* = 4). **(K)** Mitosox-Red staining was performed to estimate the mitochondrial ROS levels in cardiomyocytes. **(L)** Representative images of Mitosox-Red-stained cardiomyocytes. Scale bar reflects 100 μm. Fluorescence intensity was measured using ImageJ software (*n* = 3). **(M–P)** The expression levels of antioxidant genes SOD1 **(M)**, CAT **(N)**, MnSOD **(O)**, Prx-3 **(P)** in cardiomyocytes were measured by qPCR. GAPDH was used as an internal control (*n* = 3–6). Data are expressed as the mean ± SE. Significance of the difference between DMSO and DMSO+PE or between Hispidulin and Hispidulin+PE: ^*^p < 0.05; significance of the difference between DMSO and Hispidulin or between DMSO+PE and Hispidulin+PE: ^#^*p* < 0.05. **(Q–T)** The expression levels of antioxidant genes SOD1 **(Q)**, CAT **(R)**, MnSOD **(S)**, Prx-3(T) in mouse hearts were measured by qPCR. GAPDH was used as an internal control (*n* = 3–6). **(U)** Representative immunoblots of the effects of hispidulin on the expression of PGC-1α in mice 1 week after surgery. **(V)** Quantitative analysis of PGC-1α and GAPDH expression *in vivo* (*n* = 5). **(W)** Representative immunoblots of the effects of hispidulin on the expression of PGC-1α in mice 4 weeks after surgery. **(X)** Quantitative analysis of PGC-1α and GAPDH expression *in vivo* (*n* = 4). Data are expressed as the mean±SE. Significance of the difference between DMSO+Sham and DMSO+AB or between Hispidulin+Sham and Hispidulin+AB: ^*^p < 0.05; significance of the difference between DMSO+Sham and Hispidulin+Sham or between DMSO+AB and Hispidulin+AB: ^#^*p* < 0.05 (n represents the number of independent experiments).

The mitochondrion is the main organelle responsible for ATP synthesis and anti-oxidant defense. Dysfunctional mitochondria, which manifest with structural abnormalities, reduced ATP synthesis, and increased ROS damage, play an essential role in the development of cardiac hypertrophy. As shown in Figure 5, structurally abnormal mitochondria were found in hypertrophic mouse hearts 4 weeks after AB surgery, which manifested a reduced aspect ratio and smaller mitochondrial area (Figures 5A–C). In contrast, hispidulin treatment preserved the mitochondrial structure, with an unchanged mitochondrial aspect ratio and a mitochondrial area similar to the sham group. Besides, by evaluating ATP concentration in mouse hearts and cardiomyocytes (Figures 5D, 6A), we found that hispidulin significantly improved the impaired ATP levels resulting from AB and PE, thus indicating that hispidulin rescued mitochondrial ATP synthesis capacity in cardiac hypertrophy. Then, mitochondrial oxygen consumption rates (OCRs) were determined to evaluate the mitochondrial respiratory function of cardiomyocytes. Under normal condition, hispidulin treatment significantly improved mito-OCR, ATP-linked OCR and the mitochondrial reserve capacity. After PE treatment, mito-OCR, ATP-linked OCR were increased, but mitochondrial reserve capacity was significantly decreased. Meanwhile, hispidulin further improved mitochondrial reserve capacity under PE treatment (Figures 6B–E). Thus, it suggested that hispidulin could protect mitochondrial respiratory function in the process of cardiomyocyte hypertrophy. Moreover, mitochondrial dysfunction leads to excessive oxidative damage. To determine the ROS levels *in vivo* and *in vitro*, DHE staining was conducted to evaluate the level of ROS in mouse hearts, while DCF-DA and Mitosox-red staining were used to measure cellular and mitochondrial ROS in cardiomyocytes, respectively. We found that hispidulin reduced the cellular and mitochondrial ROS induced by AB and PE treatment (Figures 6F–L). Additionally, hispidulin significantly upregulate the mRNA expression of antioxidant enzymes, including superoxide dismutase 1 (SOD1), manganese-containing superoxide dismutase (MnSOD) and catalase (CAT) under both PE and AB treatment, and the expression of Peroxiredoxin-3(Prx-3) were barely altered, indicating that hispidulin suppressed mitochondrial and cellular oxidative stress (Figures 6M–T). Considering PPARγ coactivator-1 family of transcriptional coactivator-1α (PGC-1α) serves as a key regulator in mitochondria, the expression of PGC-1α protein was evaluated in mice 1 week and 4 weeks after surgery. As shown in Figures 6U–X, hispidulin significantly increased the expression of PGC-1α protein both in 1 week and 4 weeks after AB surgery, with mildly alternation in sham group. According to the above results, we speculated that hispidulin could improve mitochondrial dysfunction in cardiac hypertrophy.

### Hispidulin Restored Mitochondrial Fusion and Fission in the Heart

Considering mitochondrial dynamics are pivotal for mitochondrial function, we examined several mitochondrial fusion-related genes, including optic atrophy 1 (Opa1), mitofusin 1 (Mfn1) and mitofusin 2 (Mfn2), as well as fission-related genes including Dynamin-related protein1 (Drp1), Mitochondrial fission protein 1 (Fis1), Mitochondrial fission factor (Mff) *in vitro*. As shown in Supplementary Figures 1A,B, hispidulin rescued PE-impaired mitochondrial fusion and fission, with elevated levels of Mfn1, Mfn2, Opa1, Fis1, Drp1, and Mff. Similar to the findings *in vivo*, hispidulin significantly improved the protein expression of Opa1, Mfn2, and translocase of outer mitochondrial membrane 20 (Tom20) in hypertrophic hearts. Hence, our results demonstrated that hispidulin could improve the level of mitochondrial dynamics both *in vivo* and *in vitro*.

### Hispidulin Activated Sirt1 and Exert Its Cardio-Protective Effect

**Figure 7:**
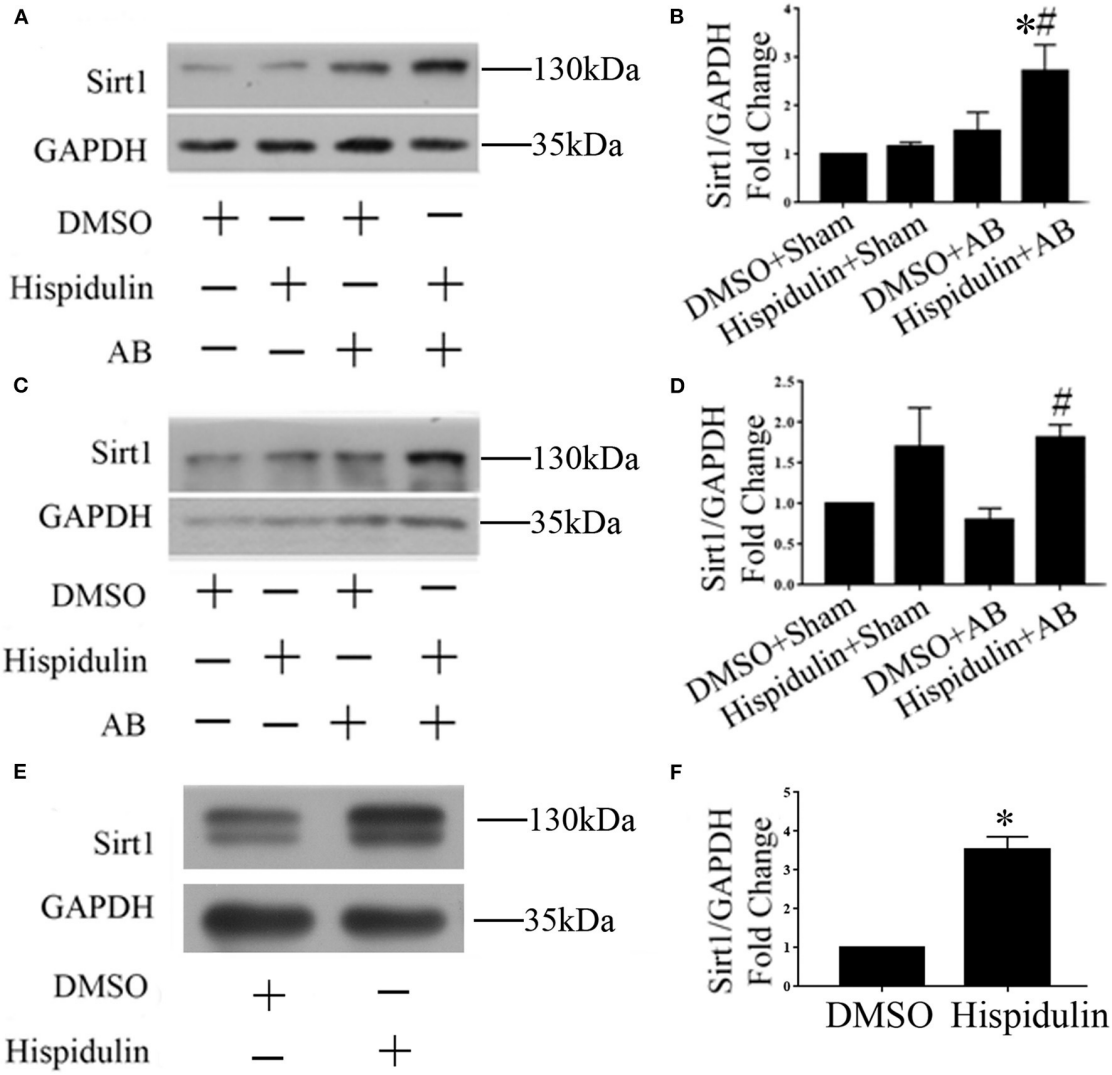
Hispidulin promoted Sirt1 expression in mouse hearts. **(A)** Representative immunoblots of the effects of hispidulin on the expression of Sirt1 in mice 1 week after surgery. **(B)** Quantitative analysis of Sirt1 and GAPDH expression *in vivo* (*n*=3). **(C)** Representative immunoblots of the effects of hispidulin on the expression of Sirt1 in mice 4 weeks after surgery. **(D)** Quantitative analysis of Sirt1 and GAPDH expression *in vivo* (*n* = 5). Data are expressed as the mean ± SE. Significance of the difference between Hispidulin+Sham and Hispidulin+AB: ^*^p < 0.05; significance of the difference between DMSO+AB and Hispidulin+AB: ^#^*p* < 0.05. **(E)** Representative immunoblots of the effects of hispidulin on the expression of Sirt1 in isolated adult mouse cardiomyocytes 24 h after hispidulin treatment. **(F)** Quantitative analysis of Sirt1 and GAPDH expression *in vitro* (*n* = 3). Data are expressed as the mean ± SE. Significance of the difference between DMSO and Hispidulin: ^*^p < 0.05 (n represents the number of independent experiments).

**Figure 8:**
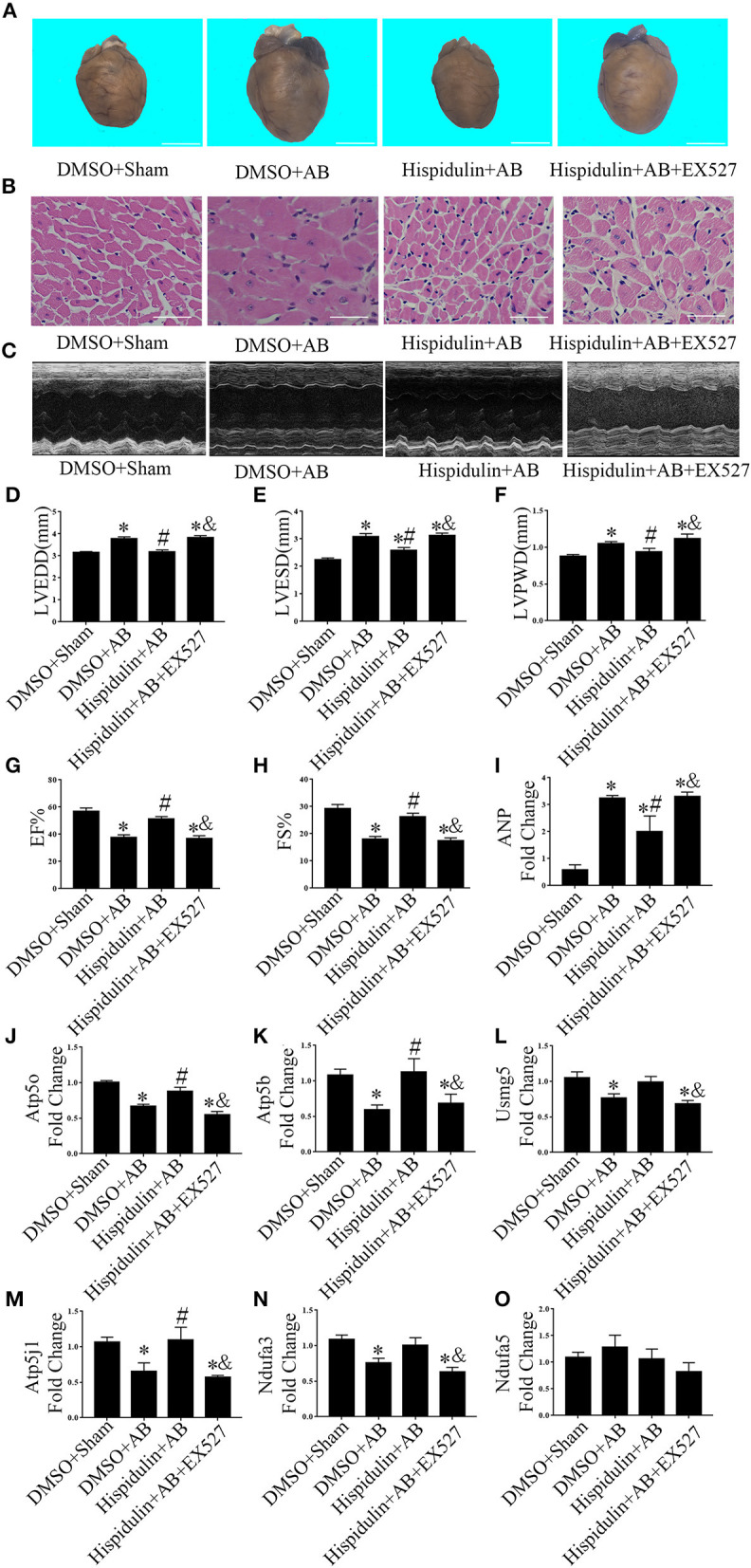
EX527 blocked the cardioprotective effects of hispidulin on mice. EX527 were injected intraperitoneally 1 day before surgery through 4 weeks after surgery. **(A)** Images of hearts harvested 4 weeks after surgery. The scale bar refers to 2 mm. **(B)** H&E staining of heart paraffin sections 4 weeks after surgery. Scale bar refers to 50 μm. **(C)** Echocardiographic images of the hearts in each group. **(D–H)** Histograms of LVEDD **(D)**, LEVSD **(E)**, LVPWD **(F)**, EF% **(G)** and FS% **(H)** (*n* = 5–16). **(I)** The mRNA expression of ANP was detected by qPCR. GAPDH was used as an internal control (*n* = 4). **(J–O)** The mRNA expressions of ETC complex subunits including Atp5o **(J)**, Atp5b **(K)**, Usmg5 **(L)**, Atp5j1 **(M)**, Ndufa3 **(N)** and Ndufa5 **(O)** were detected by qPCR. GAPDH was used as an internal control (*n* = 4). Data are expressed as the mean ± SE. Significance of the difference between DMSO+Sham and other 3 groups, respectively: ^*^p < 0.05; significance of the difference between DMSO+AB and Hispidulin+AB: ^#^*p* < 0.05; significance of the difference between Hispidulin+AB and Hispidulin+AB+EX527: ^&^*p* < 0.05 (n represents the number of independent experiments).

Sirt1 is a potent regulator of mitochondrial function, and previous studies have demonstrated that Sirt1 regulates mitochondrial function by modulating PGC-1α (17, 18). We speculated that Sirt1 might be a downstream target of hispidulin, and the alternation of Sirt1 protein was detected in mice in 1 week and 4 weeks post-operatively. As shown in Figures 7A–D, hispidulin elevated the protein expression of Sirt1 in both 1 week and 4 weeks after AB surgery, compared with mice in the DMSO group. Besides, we investigated the expression of Sirt1 on adult mouse cardiomyocytes treated with DMSO or hispidulin. We found that hispidulin increased the Sirt1 expression up to 3.53 fold under basal condition (Figures 7E,F). Intending to clarify the role of Sirt1 as a downstream target of hispidulin, our experiment blocked Sirt1 pharmacologically in mice. EX527, Sirt1-specific inhibitor, was injected intraperitoneally 1 day before surgery through 4 weeks after surgery. As Figure 8 shows, the cardioprotective effects of hispidulin were mostly abolished by injection of EX527, as evidenced by more pronounced elevation of HW/BW and HW/TL, increased cross-sectional area of cardiomyocytes, and upregulation of hypertrophic biomarker ANP in the hearts of mice that received EX527, compared with those that received hispidulin treatment alone after pressure overload (Figures 8A,B,I and Table 2). Echocardiography illustrated that the hearts which received EX527 injection had more severe hypertrophic morphology and cardiac dysfunction, manifesting elevated LVPWD, LVEDD, and LVESD as well as decreased FS% and EF% than the hearts that merely received hispidulin treatment after AB (Figures 8C–H). Besides, the expression of mitochondrial oxidative phosphorylation complex subunits was also evaluated. Although Ndufa5 expression was unaltered, the expression levels of Atp5o, Atp5b, Usmg5, Atp5j1, Ndufa3 were decreased by EX527, which consequently revealed that EX527 blocked the mitochondrial protective effect of hispidulin (Figures 8J–O). Thus, our findings suggest that Sirt1 might be a downstream effector of hispidulin in regulating cardiac hypertrophy.

In addition, AMPK has been reported to regulate the activation of Sirt1 and its downstream targets, such as PGC-1α to maintain mitochondrial energy production capacity (19), we also evaluated the expression level of AMPK in our present study. As shown in Supplementary Figure 2, hispidulin increased the phosphorylation level of AMPK after AB surgery 1 week after surgery, but the alternation became insignificant in 4 weeks after surgery. To verify the influence of hispidulin on the activation of AMPK, we detected the expression of AMPK on adult mouse cardiomyocytes treated with the DMSO or hispidulin for 24 h. Results showed that hispidulin increased phospho-AMPK up to 4.26 fold compared with the DMSO group. The above results suggested that Sirt1 is at least partly involved in the regulation of hispidulin on cardiac hypertrophy, and AMPK could be a potential upstream of it.

## Discussion

Mitochondrial dysfunction, including energy depletion and mitochondrial oxidative stress, as well as reduced mitochondrial dynamics and structural abnormalities, contributes to the development of cardiac hypertrophy. In this study, we initially demonstrated that hispidulin alleviated pressure overload and PE-induced cardiac hypertrophy. Hispidulin prevents mitochondria from dysfunction and oxidative damage in response to hypertrophy stimuli, which include maintaining mitochondrial respiratory function and ATP synthesis capability, promoting gene expression of ETC complexes and energy transduction systems, and renormalized mitochondrial morphology. Meanwhile, we discovered that Sirt1 blockage diminished the protective effect of hispidulin on cardiac hypertrophy, which indicated that Sirt1 might interact with hispidulin correspondingly in regulating cardiac hypertrophy.

Hispidulin, a natural flavonoid compound, can penetrate the cell membrane to achieve various biological functions due to its hydrophobicity. An increasing number of studies have shown that hispidulin has antioncogenic, antioxidative, antiapoptotic, antithrombotic, and antiproliferative effects (12, 20–22). Hispidulin reduces UV-mediated oxidative damage by activating Nrf2 (12). Wu et al. found that hispidulin regulates lipid metabolism by directly activating PPARα (10). In addition, hispidulin activates PPARγ through the activation of AMPK and ERK signaling pathways to inhibit hepatocellular carcinoma growth and metastasis both *in vitro* and *in vivo* (11). Benefit from the features of hispidulin mentioned above, we hypothesized that hispidulin might play a protective role against cardiac hypertrophy. With pressure overload and PE-induced cardiac hypertrophic model, we found that hispidulin could significantly attenuate the thickness and dilation of the left ventricle, fetal gene re-expression, pulmonary edema, and improve cardiac function. Furthermore, an *in vitro* study confirmed that hispidulin inhibited PE-induced cardiomyocyte hypertrophy. Those findings suggested that hispidulin functions as an effective agent protecting against cardiac hypertrophy and dysfunction. However, we found that hispidulin slightly reduced cell viability of cardiomyocytes under physiological conditions. Previous studies have found that hispidulin induced a sharply decrease of cell viability in tumor cells by inducing apoptosis while hispidulin could also protect neural cells against bupivacaine-induced injury by its anti-apoptosis effect (13, 22). The two-sided pharmacological property of hispidulin might partly result from this observation.

In the development of cardiac hypertrophy, insufficient energy aggravates cardiac dysfunction. The mitochondrion is the vital organelle for energy production, and its function is impaired in hypertrophic hearts (23). In rat with cardiac systolic dysfunction induced by aortic constriction, limited mitochondrial respiratory function, smaller mitochondrial size, reduced mitochondrial volume density, and decreased levels of ETC proteins were observed (24). Evidence has revealed that there is a significant association between mitochondrial dysfunction and heart failure (25). Improving mitochondrial function could suppress the deterioration of cardiac function. A number of studies have evaluated the function of natural compounds on mitochondria. Caffeic acid ethanolamide suppresses mitochondrial bioenergetic impairment and oxidative stress in isoproterenol-induced cardiac hypertrophy (26). Honokiol treatment blocks and reverses cardiac hypertrophy by improving the mitochondrial oxygen consumption rate and reducing ROS synthesis in mice (27). Choline ameliorates cardiac hypertrophy by improving mitochondrial metabolic remodeling (28). In a diabetic cardiomyopathy mouse model, resveratrol improved cardiac function by improving mitochondrial biogenesis and function (29). In our present study, RNA-sequencing analysis revealed that hispidulin reversed the AB surgery induced decrease in the expression of mitochondrial oxidative phosphorylation complexes subunits. Meanwhile, transmission electron microscopy showed that structural abnormalities of mitochondria were undetectable after hispidulin treatment, indicating that the improvement of impaired mitochondria could be attributed to the protective effect of hispidulin against cardiac hypertrophy. Based on our present findings, the protective effects of hispidulin on mitochondria were explored in four aspects. First, hispidulin elevated mitochondrial metabolic genes including ETC complex subunits. Besides, hispidulin improved ATP concentration in hypertrophic cardiac tissues and cardiomyocytes and normalized mitochondrial structural disorder in mouse hearts. Those effects indicated hispidulin could maintain the mitochondrial energy production capacity. Second, hispidulin treatment significantly improved mitochondrial reserved respiratory capacity, which is defined as the ability of mitochondrion to increase its ATP production, no matter with or without PE treatment. Under physiological conditions, mitochondrial ATP production can fulfill the cellular energy demand, but when energy requirement increases, the mitochondrion is of necessity to increase its ATP synthesis capacity to ensure sufficient energy supply. Reduced mitochondrial reserve respiratory capacity will lead to insufficient energy supply to cells and cell death ultimately (30). Whereas, hispidulin could increase mitochondrial reserved respiratory capacity to ensure energy supply. Third, hispidulin reduced cellular and mitochondrial oxidative damage benefit from an elevation in the expressions of antioxidants (SOD1, catalase, and MnSOD) and a reduction of ROS emission from mitochondria, as shown by Mitosox-Red staining. Briefly, oxidative damage is characterized by excessive ROS generated from inefficient ETC and impaired antioxidative defense. In addition to improving ETC, hispidulin inhibits ROS damage by maintaining the function of the antioxidative enzymatic defense system in mitochondria. Last, the depression of mitochondrial dynamics was also rescued by hispidulin. Fusion and fission, as the bridge linking between mitochondrial biogenesis and mitophagy, are essential to maintain the function of mitochondria (31). Fusion facilitates mitochondrial DNA damage repair, and fission enables damaged mitochondrial components to separate from mitochondria and then undergo degradation (32, 33). Previous studies confirmed that specific deprivation of either fusion and fission related genes in the heart resulted in dilated cardiomyopathy and heart failure (34). Accordingly, the protection of hispidulin against cardiac hypertrophy and heart failure could result from the alleviation of mitochondrial function.

Sirtuins (Sirts) represent a highly evolutionarily conserved family of NAD-dependent deacetylases. Seven members of this family (Sirt1–7) have been identified (17). Sirt1, the most studied member of this family, is enriched in cardiomyocytes and maintains mitochondrial function under physical conditions and stress stimuli (35, 36). The deletion of Sirt1 in mice causes the development of dilated cardiomyopathy, and subsequently being more vulnerable to cardiac injury induced by ischemia/reperfusion (I/R). This phenomenon could be explained by a reduction of the transcription of antioxidative enzymes which include superoxide dismutase and catalase (37). The upregulation of Sirt1 induced by resveratrol protects the heart against isoproterenol-induced cardiac hypertrophy (29). Besides, Sirt1 has been proved to upregulate PPARs and PGC-1α activity, which modulate mitochondrial energy transduction, ATP synthesis, and biogenesis (36). In the present study, accompanied by an alleviated hypertrophic phenotype, hispidulin was able to upregulate Sirt1 expression in the presence of pressure overload-induced cardiac hypertrophy as well as in isolated adult mouse cardiomyocytes. Moreover, pharmacological inhibition of Sirt1 abolished most of the protective effects of hispidulin, represented as aggravated cardiac hypertrophy, impaired cardiac function, and reduced expression of ETC subunits. Those findings indicated that Sirt1 could be a downstream target of hispidulin. Finally, AMPK has been proven to be an upstream regulator of Sirt1 (19), meantime our findings demonstrated that hispidulin increased the level of AMPK phosphorylation. Hence, the protective effect of hispidulin might due to the upregulation of Sirt1 through activating AMPK.

## Limitations

The cardioprotective effect of hispidulin has been observed on AB- and PE-induced cardiac hypertrophy model. However, this study has potential limitations. First, the mechanism of reduced cell viability of hispidulin in cardiomyocytes under physiological conditions was still unclear. Besides, it has not been fully elucidated whether hispidulin exerts its effects via other targets except activating Sirt1. Finally, the exact mechanism of hispidulin regulating Sirt1 still needs to be further investigated.

## Conclusion

In summary, our study demonstrated that hispidulin prevents AB- and PE-induced pathological cardiac hypertrophy by maintaining mitochondrial function. Mechanically, Sirt1 could be the pivotal therapeutic target involved in the protection of hispidulin. These findings provide novel evidence that hispidulin might be a promising treatment for cardiac hypertrophy and heart failure.

## Data Availability Statement

The datasets generated for this study can be found in online repositories. The names of the repository/repositories and accession number(s) can be found at: https://www.ncbi.nlm.nih.gov/, GSE154798.

## Ethics Statement

The animal study was reviewed and approved by Ethics Committee of ZSSOM on Laboratory Animal Care.

## Author Contributions

All authors contributed listed in this paper contributed to the study. YW and ZX performed the almost of experiments, analyzed the data, and wrote the manuscript. NJ, ZW, and RX designed the study and acquired data. BD, WF, and GD participated in the design the study and analyzed the data. CC, JL, and HC performed parts of experiments and acquired the data. ZY, RF, MC, and JZ performed parts of experiments and analyzed the data. YD and CL participate in the design the study and revised the manuscript. All authors read the final version of manuscript and approved.

## Conflict of Interest

The authors declare that the research was conducted in the absence of any commercial or financial relationships that could be construed as a potential conflict of interest.

## Supplementary Material

Supplementary Figure 1 Hispidulin restored mitochondrial dynamics. **(A)** Hispidulin increased mitochondrial fusion after PE treatment, with elevated expressions of mitochondrial fusion related genes including Mfn1, Mfn2, Opa1 (*n* = 3 and 4). **(B)** Hispidulin increased mitochondrial fission after PE treatment, with elevated expressions of mitochondrial fission related genes Fis1, Drp1, and Mff (*n* = 3 and 4). Significance of the difference between DMSO and DMSO+PE or between Hispidulin and Hispidulin+PE: ^*^*p* < 0.05; significance of the difference between DMSO and Hispidulin or between DMSO+PE and Hispidulin+PE: ^#^*p* < 0.05. **(C)** Representative of the effects of hispidulin on the expression of Mfn2, Opa1, and Tom20 in mice 4 weeks after surgery. Quantitative analysis of Mfn2 **(D)**, Opa1 **(E)**, Tom20 **(F)** expressions *in vivo* (*n* = 3–5). GAPDH and Vinculin was used as an internal control. Data are expressed as the mean ± SE. Significance of the difference between DMSO+Sham and DMSO+AB: ^*^*p* < 0.05; significance of the difference between DMSO+Sham and Hispidulin+Sham or DMSO+AB and Hispidulin+AB: ^#^*p* < 0.05 (n represents the number of independent experiments).

Supplementary Figure 2 Hispidulin promoted phosphorylated AMPK expression in mouse hearts. **(A)** Representative immunoblots of the effects of hispidulin on the expression of AMPK in mice 1 week after surgery. **(B)** Quantitative analysis of phosphorylated and total AMPK expression *in vivo* (*n* = 3). **(C)** Representative immunoblots of the effects of hispidulin on the expression of AMPK in mice 4 weeks after surgery. **(D)** Quantitative analysis of phosphorylated and total AMPK expression *in vivo* (*n* = 3). Data are expressed as the mean ± SE. Significance of the difference between DMSO+Sham and DMSO+AB: ^*^*p* < 0.05; significance of the difference between DMSO+AB and Hispidulin+AB: ^#^*p* < 0.05. **(E)** Representative immunoblots of the effects of hispidulin on the expression of AMPK in isolated mouse cardiomyocytes 24 h after hispidulin treatment. **(F)** Quantitative analysis of phosphorylated and total AMPK expression *in vitro* (*n* = 3). Data are expressed as the mean ± SE. Significance of the difference between DMSO and Hispidulin: ^*^*p* < 0.05 (n represents the number of independent experiments).
