# Supplementary figures and images for: Hispidulin Attenuates Cardiac Hypertrophy by Improving Mitochondrial Dysfunction
Source: Front Cardiovasc Med. 2020 Nov 26;7:582890. doi: 10.3389/fcvm.2020.582890 (PMC7726192; doi:10.3389/fcvm.2020.582890)

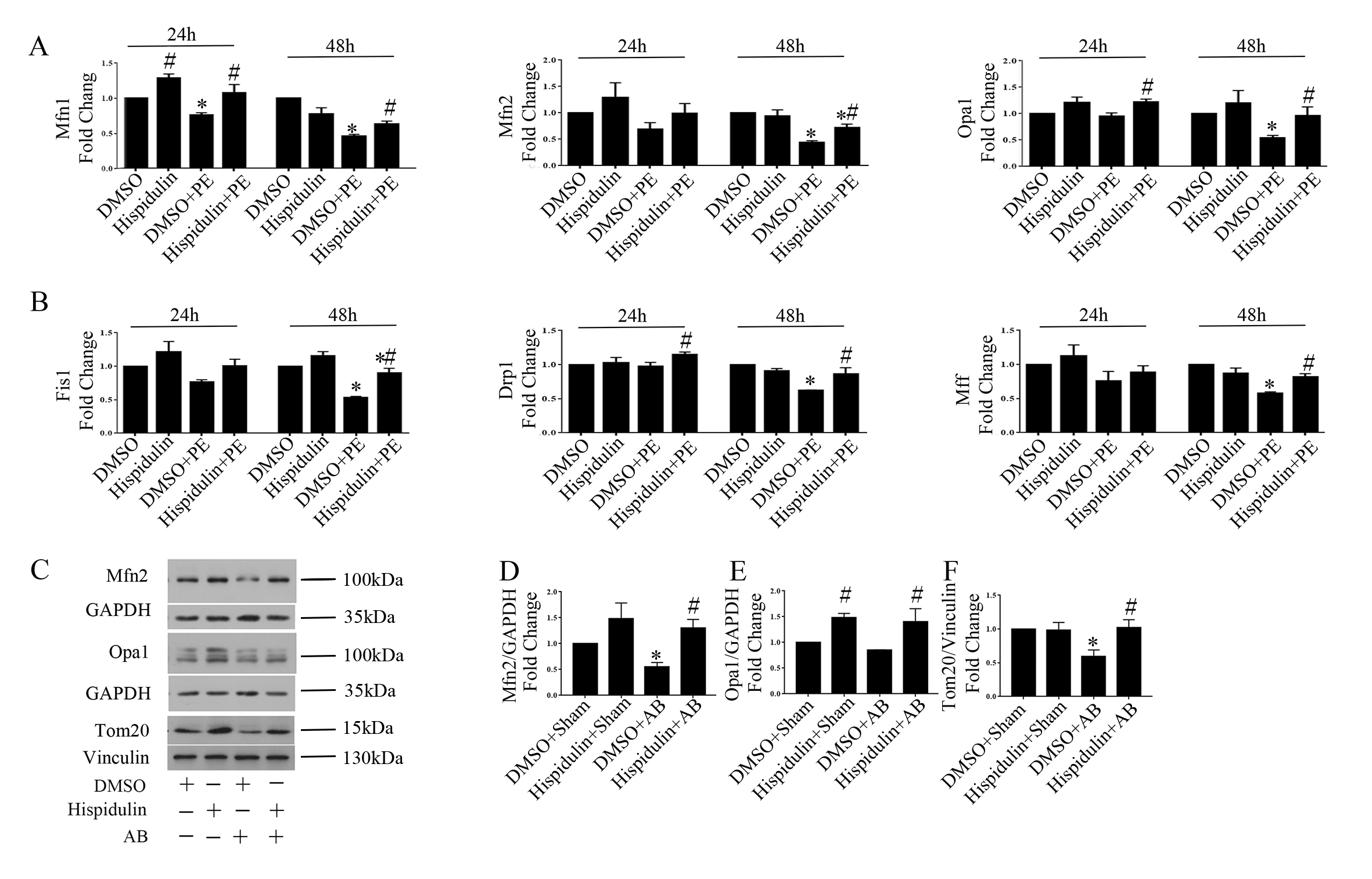

Supplement: Supplementary Figure 1 — Hispidulin restored mitochondrial dynamics. (A) Hispidulin increased mitochondrial fusion after PE treatment, with elevated expressions of mitochondrial fusion related genes including Mfn1, Mfn2, Opa1 (n = 3 and 4). (B) Hispidulin increased mitochondrial fission after PE treatment, with elevated expressions of mitochondrial fission related genes Fis1, Drp1, and Mff (n = 3 and 4). Significance of the difference between DMSO and DMSO+PE or between Hispidulin and Hispidulin+PE: *p < 0.05; significance of the difference between DMSO and Hispidulin or between DMSO+PE and Hispidulin+PE: #p < 0.05. (C) Representative of the effects of hispidulin on the expression of Mfn2, Opa1, and Tom20 in mice 4 weeks after surgery. Quantitative analysis of Mfn2 (D), Opa1 (E), Tom20 (F) expressions in vivo (n = 3–5). GAPDH and Vinculin was used as an internal control. Data are expressed as the mean ± SE. Significance of the difference between DMSO+Sham and DMSO+AB: *p < 0.05; significance of the difference between DMSO+Sham and Hispidulin+Sham or DMSO+AB and Hispidulin+AB: #p < 0.05 (n represents the number of independent experiments). [file Image_1.TIF]

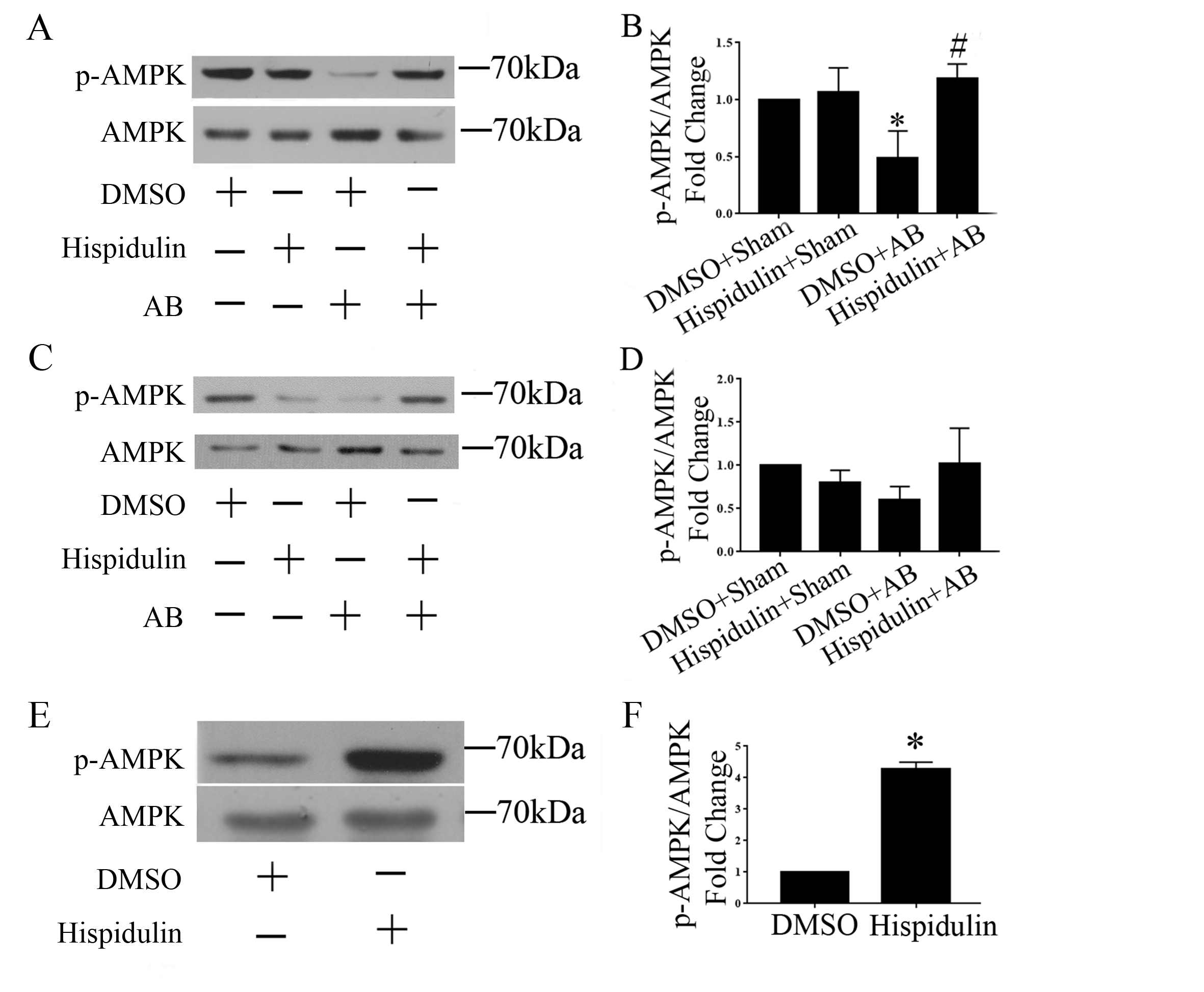

Supplement: Supplementary Figure 2 — Hispidulin promoted phosphorylated AMPK expression in mouse hearts. (A) Representative immunoblots of the effects of hispidulin on the expression of AMPK in mice 1 week after surgery. (B) Quantitative analysis of phosphorylated and total AMPK expression in vivo (n = 3). (C) Representative immunoblots of the effects of hispidulin on the expression of AMPK in mice 4 weeks after surgery. (D) Quantitative analysis of phosphorylated and total AMPK expression in vivo (n = 3). Data are expressed as the mean ± SE. Significance of the difference between DMSO+Sham and DMSO+AB: *p < 0.05; significance of the difference between DMSO+AB and Hispidulin+AB: #p < 0.05. (E) Representative immunoblots of the effects of hispidulin on the expression of AMPK in isolated mouse cardiomyocytes 24 h after hispidulin treatment. (F) Quantitative analysis of phosphorylated and total AMPK expression in vitro (n = 3). Data are expressed as the mean ± SE. Significance of the difference between DMSO and Hispidulin: *p < 0.05 (n represents the number of independent experiments). [file Image_2.TIF]
